# Quality indicators for ambulatory care for older adults with diabetes and comorbid conditions: A Delphi study

**DOI:** 10.1371/journal.pone.0208888

**Published:** 2018-12-13

**Authors:** Yelena Petrosyan, Jan M. Barnsley, Kerry Kuluski, Barbara Liu, Walter P. Wodchis

**Affiliations:** 1 Clinical Epidemiology, The Ottawa Hospital Research Institute, Ottawa, Canada; 2 Institute of Health Policy, Management and Evaluation, University of Toronto, Toronto, Canada; 3 Lunenfeld Tanenbaum Research Institute, Sinai Health System, Toronto, Canada; 4 Sunnybrook Health Sciences Centre, University of Toronto, Toronto, Canada; 5 Institute for Clinical Evaluative Sciences, Toronto, Canada; 6 Toronto Rehabilitation Institute, Toronto, Canada; Florida International University, UNITED STATES

## Abstract

**Background:**

An increasing number of people are living with multiple chronic conditions and it is unclear which quality indicators should be used to guide care for this population.

**Objective:**

To critically appraise and select the most appropriate set of quality indicators for ambulatory care for older adults with five selected disease combinations.

**Methods:**

A two-round web-based Delphi process was used to critically appraise and select quality of care indicators for older adults with diabetes and comorbidities. A fifteen-member Canadian expert panel with broad geographical and clinical representation participated in this study. The panel evaluated process indicators for meaningfulness, potential for improvements in clinical practice, and overall value of inclusion, while outcome indicators were evaluated for importance, modifiability and overall value of inclusion. A 70% agreement threshold was required for high consensus, and 60–69% for moderate consensus as measured on a 5-point Likert type scale.

**Results:**

Twenty high-consensus and nineteen medium-consensus process and outcome indicators were selected for assessing care for older adults with selected disease combinations, including 1) concordant (conditions with a common management plan), 2) discordant (conditions with unrelated management plans), and 3) both types. Panelists reached rapid consensus on quality indicators for care for older adults with concordant comorbid conditions, but not for those with discordant conditions. All selected indicators assess clinical aspects of care. The feedback from the panelists emphasized the importance of developing indicators related to patient-centred aspects of care, including patient self-management, education, patient-physician relationships, and patient’s preferences.

**Conclusions:**

The selected quality indicators are not intended to provide a comprehensive tool set for measuring quality of care for older adults with selected disease combinations. The recommended indicators address clinical aspects of care and can be used as a starting point for ambulatory care settings and development of additional quality indicators.

## Introduction

Evidence shows that the majority of care for adults with multiple chronic conditions is provided in ambulatory care settings such as primary care, and this is a logical setting from which to develop approaches of care to better meet the needs of this population [1, 2]. Evidence also shows that older adults are more likely than younger individuals to have comorbid chronic conditions that can be complex and difficult to manage [3, 4]. Recent research has demonstrated that that more than 90% of older adults with diabetes in Ontario had at least one comorbid condition [5]. Several studies have found that hypertension consistently appeared in the majority of the top ten condition clusters for older adults with diabetes [3, 5, 6]. Other chronic conditions that appeared in many of the clusters for older adults were arthritis, other cardiovascular conditions and mood disorders [3, 5].

Diabetes in particular is a common focus for clinical quality assessment and performance contacts in value-based programs such as Accountable Care Organizations [7]. Using quality indicators derived from administrative claims data is a common way of measuring and monitoring the quality of care and services [8]. While disease-specific indicators are important, they may not be sufficient to evaluate the appropriateness of care for people with multimorbidity [9–11]. For instance, nonsteroidal anti-inflammatory medications for pain relief from osteoarthritis would aggravate hypertension and renal disease in diabetes patients [12, 13].

Quality indicators for cases of multiple conditions should consider whether the conditions share a common management plan (concordant) or not (discordant). There are few published indicators for care for older adults with diabetes that specifically address comorbidity with concordant conditions, including hypertension and chronic ischemic heart disease [14–16]. Moreover, there are no indicators identified for care for older adults with diabetes with comorbid discordant conditions, including osteoarthritis and major depression [17, 18]. The present study addresses these gaps and aimed to: 1) critically appraise and select the most appropriate set of quality indicators for ambulatory care for older adults with five selected disease combinations that are amenable to measurement using administrative data, using a Delphi technique, including: a) concordant conditions: diabetes with comorbid hypertension and ischemic heart disease, b) discordant conditions: diabetes with comorbid osteoarthritis and depression, c) both types: diabetes with comorbid hypertension and osteoarthritis, and 2) prepare a summary of defined quality indicators by each selected disease combination. A set of selected indicators will be used for assessing the quality of care for older adults with selected disease combination in ambulatory care settings using Ontario administrative data. All provinces in Canada hold administrative data for the full population under a universal health care system that is similar to other health systems internationally including diagnoses and utilization from physician, hospital and pharmacy billing data.

## Methods

The consensus process incorporated a two-round web-based Delphi method, which took place between October 2014 and March 2015. A Delphi technique is widely used for developing quality indicators in healthcare [19]. The Delphi method is an acknowledged method to gather consensus of opinion and choice about a topic, in this case, different types of quality indicators from a selected panel [20]. It is a structured iterative process that uses repetitive administration of questionnaires to gather information [19].

### Ethics statement

Ethics approval for this study was obtained from the University of Toronto Research Ethics Board.

### Selection of conditions

Five prevalent chronic conditions among older adults were included for the purpose of this study, including diabetes, hypertension, ischemic heart disease, osteoarthritis, and depression. Depression in this study connotes major depression and dysthymia, since most clinical practice guidelines only address treatment of major depression [21]. These five chronic diseases were categorized into three groups by comorbidity type relative to diabetes [22], including concordant conditions that share a common management plan (such as diabetes with comorbid hypertension and ischemic heart disease), discordant conditions that are not directly related in the disease management plan (diabetes with comorbid osteoarthritis and diabetes with osteoarthritis and major depression), and both types (diabetes with comorbid hypertension and osteoarthritis). These five disease combinations represented most prevalent clusters of concurrent conditions across multimorbidity groupings based on the prior research results [3].

### Assembly of Delphi panel

Panelists were selected with the goal of having a representative group of experts to better reflect the variety of specialities that are involved in multimorbid patient treatment decisions. The panel consisted of geriatricians, primary care specialists, clinical pharmacists, and a clinician-researcher. There was diverse geographical representation among the panel members, with representation from various provinces of Canada, including Ontario, British Columbia and Quebec. A key component of the Delphi technique is the anonymity of the expert panel members. Thus, no panelist knew the identity of the other panel members. This allowed consensus to be reached among the panel members without the issues that may arise from peer influence.

### Questionnaire preparation

First, systematic reviews were conducted to identify a set of evidence-based and valid quality indicators for assessing care for older adults in ambulatory care settings by each disease category, including diabetes, major depression, hypertension, chronic ischemic heart disease and osteoarthritis, as well as selected disease combinations [17, 18]. The resulting indicators were then sorted into those potentially measurable with the Ontario administrative data, and those that required other sources of data. The latter group was excluded from the study.

A list of potential indicators was included in the first-round questionnaire for each selected disease combination. In particular, the initial set of candidate indicators was compiled by combining the identified indicators for care for the relevant single conditions for each disease combination, accordingly. These indicators were divided into process of care indicators, that examine how care has been provided, and outcome indicators, that attempt to describe the effects of care on the health status of patients or population [23]. The initial questionnaire with candidate indicators was pre-tested with three physicians (who were not recruited to the Delphi panel) to anticipate the average completion time, and for clarity.

Once the list of candidate panelists was formed, each person was contacted via e-mail. All candidates were sent invitations to participate which included a description of the study, its objectives, the number of Delphi rounds to be included, the promise of anonymity, as well as benefits from participation, and a confirmation of the panelist’s acceptance.

### Delphi Round 1

The panelists who confirmed participation in the study received the first-round questionnaire by electronic mail along with the appraisal tool criteria and instructions for rating. The panelists were also provided with detailed information related to the candidate indicators selected during the systematic review, including numerator/denominator, inclusion/exclusion criteria, data source, and rationale/supporting evidence. The panel was asked to rate each potential indicator, on a five-point scale, according to the appraisal criteria as adapted from the methodology for eliciting expert opinion using the Delphi technique and used in prior research [24, 25]. A score of one indicated the lowest rating and a score of five indicated the highest possible rating.

Criteria for rating process indicators included: 1) meaningfulness: whether this is a meaningful measure of the quality of care we deliver to the older adults with this disease combination, 2) potential for improvements in clinical practices: whether it is possible to improve the care that impacts this indicator in older adults with this disease combination; and 3) overall value of inclusion: considering your ratings on all dimensions, rate this process measure overall for inclusion in the context of this disease combination (S1 Table). Criteria for rating outcome indicators included: 1) importance: whether this outcome is an important indicator of the quality of care of older adults with this disease combination, 2) modifiability: whether this outcome is potentially modifiable by improvements in patient’s care, and 3) overall value of inclusion: considering your ratings on all dimensions, rate this outcome measure overall for inclusion in the context of this disease combination (S2 Table).

Consensus level was defined based on the RAND/UCLA Appropriateness Methodology agreement definition for a panel size of 15 –at least 11 of 15 panelists rated an indicator a 4 or 5, or 1 or 2 [26]. The extent of disagreement in ratings was defined by the panel’s mean absolute deviation from the median (MADM)–the average distance of the panelists’ ratings from the panel’s median rating [27]. Thus, consensus for Round I was defined based on three selection criteria: 1) panel median score of 4 or 5, or 1 or 2; 2) having at least 11 of 15 panelists (73%) rated a given indicator a 4 or 5 –“should include or must include”, or 1 or 2 –“do not include or little reason to include” [26], and 3) mean absolute deviation from the median (MADM) less or equal to 1.03 [27].

### Delphi Round 2

All panelists who had participated in Round I were sent an email with the second-round questionnaire along with the results of the first round including median panel rating for each indicator, their individual rating from the first round, the frequency distribution of all panelists ratings, as well as comments [26]. Quality indicators that received panel consensus in Round I were not represented in Round II [28]. The remaining indicators were included in Round II of the study together with the modified and/or new indicators suggested by the panelists. The panelists were asked to re-score each quality indicator using the same criteria for rating based on their own opinion and the panel responses obtained during the first round.

To be included in the final list of quality indicators, the items were selected by two levels of consensus for each selected disease combination [29]:

- High consensus: a minimum of 11 of 15 panelists (73%) rated a given indicator a 4 or 5 –“should include or must include”, or 1 or 2—“do not include or little reason to include”, and the mean absolute deviation from the median was less or equal to 1.03;
- Moderate consensus: 9 or 10 of 15 panelists (60–67%) rated a given indicator a 4 or 5 –“should include or must include”, or 1 or 2—“do not include or little reason to include”, and the mean absolute deviation from the median was less or equal to 1.03.

## Results

The characteristics of the panelists who participated in the study/non-respondents are presented in Table 1. Of 30 panelists contacted to participate in the Delphi study, 15 (50%) accepted: six geriatricians, four primary care specialists, two general internists, two clinical pharmacists, and a clinician-researcher. All participants completed both rounds of the Delphi survey.

**Table 1:** Main characteristics of the expert panel.

Characteristics	Expert panel (n = 15)	Non-respondents (n = 15)
**Sex, n (%)**		
Male	9 (60%)	10 (66%)
Female	6 (40%)	5 (34%)
**Specialty, n (%)**		
Geriatrics	6 (40%)	6 (40%)
Primary care	4 (27%)	8 (53%)
General internal medicine	2 (13%)	0
Clinical pharmacy	2 (13%)	0
Clinician-researcher	1 (7%)	1 (7%)
**Provinces of Canada, n (%)**		
Ontario	11 (73%)	11 (73%)
British Columbia	2 (13%)	4 (27%)
Quebec	2 (13%)	0

### Delphi Round I

Of the 66 potential quality indicators for ambulatory care for older adults with five selected disease combinations, first level of consensus was reached on 17 indicators (at least 70% rated an indicator a 4 or 5, and MADM was less or equal to 1.03). These indicators included 4 indicators for care for older adults with diabetes and hypertension, 5 indicators for care for older adults with diabetes, hypertension and ischemic heart disease, 2 indicators for care for older adults with diabetes and osteoarthritis, 2 indicators for care for older adults with diabetes, osteoarthritis and major depression, and 4 indicators for care for older adults with diabetes, osteoarthritis and hypertension (S3 Table). The indicators that reached a first-round consensus were not included in the second-round questionnaire.

### Delphi Round II

Adjustments were made prior to the second round taking into account the suggestions and comments of the panelists. Eighty five indicators were evaluated during the second Delphi round, including 49 indicators that did not reach a first-round consensus, including those that were modified, and 36 additional indicators that were suggested by the panel, which were considered as “new indicators”. The requirement that indicators could be measured using Ontario administrative data was applied again.

After the first round, the following indicators were modified as “negative indicator” based on panelists’ identifications as indicators of poor performance: 1) use of non-selective NSAIDs or use of cox-selective NSAIDs for care of older diabetes patients with comorbid osteoarthritis, as well as for care for older diabetes patients with comorbid osteoarthritis and hypertension, 2) use of non-selective NSAIDs, use of cox-selective NSAIDs, use of tetracyclic antidepressant, benzodiazepines, gaba receptor agonists, or monoamine oxidase inhibitors for care of older diabetes patients with comorbid osteoarthritis and major depression. The indicator “use of tricyclic antidepressants” was excluded from the group of “negative” indicators for older diabetes patients with comorbid osteoarthritis and major depression and presented as a positive indicator in the second round based on panelists’ suggestions.

In the second round, indicators were evaluated using the same assessment criteria as in Round I. Of the 85 indicators, 11 indicators reached the high level of consensus to be included in the final list of indicators (at least 70% rated an indicator a 4 or 5 and MADM was less or equal to 1.03, one indicator reached the high level of consensus not to be included in the final list (at least 70% rated an indicator a 1 or 2 and MADM was less or equal to 1.03), 23 indicators reached the moderate level of consensus to be included in the final list (60–69% rated an indicator as 4 or 5, and MADM was less or equal to 1.03), and three indicators reached the moderate level of consensus not to be included in the final list (60–69% rated an indicator as 1 or 2, and MADM was less or equal to 1.03). Results are presented in S4 Table.

### Final indicators

After two rounds, the list of 47 final indicators was compiled, including: 1) 6 process and 5 outcome indicators for care for older adults with diabetes and hypertension, 2) 6 process and 6 outcome indicators for care of older adults with diabetes, hypertension and ischemic heart disease, 3) 4 process and 3 outcome indicators for care for older adults with diabetes and osteoarthritis, 4) 6 process and 3 outcome indicators for care for older adults with diabetes, osteoarthritis and major depression, and 5) 5 process and 3 outcome indicators for care for older adults with diabetes, osteoarthritis and hypertension. Results are presented in Table 2.

**Table 2:** Final list of quality indicators.

**Quality indicators for care for older adults with diabetes and hypertension**			
**Type**	**Indicator**	**Overall value of inclusion** Median score	**Consensus level**
**Process**	*HbA_1c_ testing every 6 months	4	High consensus
	Eye examination every 1–2 years	5	High consensus
	Microalbumin testing once per year	4	Moderate consensus
	Serum creatinine test (with eGFR)	4	High consensus
	Use of hypoglycemic drugs	4	High consensus
	Use of **ACE inhibitors or ARBs	4	High consensus
**Outcome**	Hospital admission rate for diabetes long-term complications	4	Moderate consensus
	Hospital admission rate for diabetes short-term complications	4	High consensus
	Lower-extremity amputation rate	4	High consensus
	Cardiovascular mortality rate	4	Moderate consensus
	Ocular complications due to diabetes	4	High consensus
**Quality indicators for care for older adults with diabetes, hypertension and ischemic heart disease**			
**Type**	**Indicator**	**Overall value of inclusion** Median score	**Consensus level**
**Process**	*HbA_1c_ testing every 6 months	4	High consensus
	Eye examination every 1–2 years	5	High consensus
	Microalbumin testing once per year	4	Moderate consensus
	Antiplatelet therapy	4	Moderate consensus
	**Use of ACE inhibitors or ARBs therapy	4	High consensus
	Statin therapy	4	High consensus
**Outcome**	Hospital admission rate for diabetes long-term complications	4	Moderate consensus
	Hospital admission rate for diabetes short-term complications	4	High consensus
	Lower-extremity amputation rate	4	Moderate consensus
	Cardiovascular mortality rate	4	High consensus
	Hospital admission for heart failure	4	Moderate consensus
	ED visits for diabetes short-term complications	4	Moderate consensus
**Quality indicators for care for older adults with diabetes and osteoarthritis**			
**Type**	**Indicator**	**Overall value of inclusion** Median score	**Consensus level**
**Process**	*HbA_1c_ testing every 6 months	4	Moderate consensus
	Eye examination every 1–2 years	5	High consensus
	Microalbumin testing once per year	4	Moderate consensus
	***Non-selective NSAID therapy “negative indicator”	4	Moderate consensus
**Outcome**	Hospital admission rate for diabetes short-term complications	4	High consensus
	Lower-extremity amputation rate	4	High consensus
	Cardiovascular mortality rate	4	Moderate consensus
**Quality indicators for care for older adults with diabetes, osteoarthritis and major depression**			
**Type**	**Indicator**	**Overall value of inclusion** Median score	**Consensus level**
**Process**	*HbA_1c_ testing every 6 months	4	Moderate consensus
	Eye examination every 1–2 years	5	High consensus
	Microalbumin testing once per year	4	Moderate consensus
	Interval between ****SSRIs and monoamine oxidase therapy	4	High consensus
	***Non-selective NSAIDs therapy -“negative indicator”	4	Moderate consensus
	Use of tetracyclic antidepressants, benzodiazepines, gaba receptor agonists, or monoamine oxidase inhibitors—“negative indicator”	4	Moderate consensus
**Outcome**	Hospital admission rate for diabetes long-term complications	4	Moderate consensus
	Hospital admission rate for diabetes short-term complications	4	Moderate consensus
	Cardiovascular mortality rate	4	High consensus
**Quality indicators for care for older adults with diabetes, osteoarthritis and hypertension**			
**Type**	**Indicator**	**Overall value of inclusion** Median score	**Consensus level**
**Process**	*HbA_1c_ testing every 6 months	4	Moderate consensus
	Eye examination every 1–2 years	5	High consensus
	Microalbumin testing once per year	4	Moderate consensus
	**Use of ACE inhibitors or ARBs therapy	4	High consensus
	Non-selective ***NSAID therapy “negative indicator”	4	Moderate consensus
**Outcome**	Hospital admission rate for diabetes short-term complications	4	Moderate consensus
	Hospital admission rate for diabetes long-term complications	4	Moderate consensus
	Cardiovascular mortality rate	4	High consensus

*HbA1c testing = glycated hemoglobin testing

**ACE inhibitors = angiotensin converting enzyme inhibitors; ARBs = angiotensin receptor blockers

*** NSAIDs therapy = non-steroidal anti-inflammatory drugs

****SSRIs = selective serotonin re-uptake inhibitors

After two rounds, 53 quality indicators were not included in the final list, including: 1) 49 quality indicators that did not reach consensus, and 2) 4 quality indicators reached consensus not to be included in the final list (Table 3).

**Table 3:** Quality indicators that were not included in the final list after two-round Delphi study.

**Quality indicators for care for older adults with diabetes and hypertension**	
**Indicator**	**Reason**
*LDL-cholesterol testing once per year	No consensus
Statin therapy	No consensus
Antiplatelet therapy	No consensus
Baseline electrocardiography	No consensus
**MRI of head/heart	Consensus to reject
All-cause mortality	No consensus
Urinary/skin/soft tissue infections	No consensus
**Quality indicators for care for older adults with diabetes, hypertension and chronic ischemic heart disease**	
**Indicator**	**Reason**
*LDL- cholesterol testing once per year	No consensus
Beta-blockers therapy	No consensus
All-cause mortality rate	No consensus
Bariatric surgery rate	Consensus to reject
**Quality indicators for care for older adults with diabetes and osteoarthritis**	
*LDL-cholesterol testing once per year	No consensus
Acetaminophen as first-line therapy	No consensus
Non-selective NSAIDs in combination with misoprostol/proton pump inhibitors	No consensus
Cox-selective ***NSAID therapy “negative indicator”	No consensus
Use of topical ***NSAIDs	No consensus
Statin therapy	No consensus
Use of opioids	No consensus
****Use of ACE inhibitors	No consensus
Referral for home care	No consensus
Hospital admission rate for diabetes long-term complications	No consensus
All-cause mortality	No consensus
Joint replacement therapy	No consensus
ED visits/hospital admissions for fall	No consensus
**Quality indicators for care for older adults with diabetes, osteoarthritis and major depression**	
**Indicator**	**Reason**
*LDL- cholesterol testing once per year	No consensus
Use of acetaminophen as first-line therapy	No consensus
Non-selective NSAIDs therapy in combination with misoprostol or proton pump inhibitors	No consensus
At least 3 months antidepressant treatment	No consensus
At least 6 months antidepressant treatment	No consensus
Cox-selective NSAID therapy “negative indicator”	No consensus
*****Use of SSRI or SNRI	Consensus to reject
Use of tricyclic antidepressants	No consensus
Use of topical NSAIDs	No consensus
Use of opioids	No consensus
Referral for home care	No consensus
Lower-extremity amputation rate	No consensus
ED visits/hospital admissions for falls	No consensus
Hospital admission for depression	No consensus
All-cause ED visits	No consensus
Joint replacement rate	No consensus
**Quality indicators for care for older adults with diabetes, osteoarthritis and hypertension**	
**Indicator**	**Reason**
*LDL- cholesterol testing once per year	No consensus
Statin therapy	No consensus
Beta-blocker therapy	Consensus to reject
Antiplatelet therapy	No consensus
Acetaminophen as first-line therapy	No consensus
Non-selective NSAID in combination with misoprostol or proton pump inhibitors	No consensus
***Cox-selective NSAID therapy “negative indicator”	No consensus
Use of topical NSAIDs	No consensus
Referral for home care	No consensus
Lower-extremity amputation rate	No consensus
All-cause mortality	No consensus
Joint replacement therapy	No consensus
End-stage renal disease	No consensus

*LDL-cholesterol = low-density lipoprotein cholesterol

**MRI of head/heart = magnetic resonance imaging

*** NSAIDs therapy = non-steroidal anti-inflammatory drugs

****ACE inhibitors = angiotensin converting enzyme inhibitors; ARBs = angiotensin receptor blockers

*****SSRIs = selective serotonin re-uptake inhibitors and SNRI = Serotonin and Norepinephrine Reuptake Inhibitors

## Discussion

This two-round Delphi study identified a set of 47 quality indicators for care for older adults with five selected disease combinations in ambulatory care settings, which are accessible through Ontario administrative data. To our knowledge this study is unique in its focus on developing quality indicators for care for older adults with multiple chronic conditions and listing potentially harmful indicators.

The panel reached rapid consensus on quality indicators for care for older adults with concordant comorbid conditions, but it was more complicated for care for patients with discordant conditions or both types of conditions. This finding may reflect the fact that clinical guidelines do provide a few recommendations for care of patients with diabetes with comorbid hypertension and ischemic heart disease, such as use of antihypertensive drugs, antiplatelet therapy, or use of statins [30]. Conversely, there are no guideline recommendations regarding the care of patients with diabetes with comorbid major depression and osteoarthritis.

We observed several inconsistencies between guideline recommendations and the Expert Panel members’ opinions, for example on testing and its frequency, as well as some pharmacological treatment recommendations in older adults with selected five disease combinations. For example, the annual LDL-cholesterol testing wasn’t included in the list of indicators for care for older adults with any of the selected disease combinations. The panelists mentioned that “LDL-C testing becomes less important in older people, especially without a measure of frailty”. The majority of panelists suggested excluding use of tricyclic antidepressants from the list of “negative” indicators for care for older adults with diabetes comorbid with depression and osteoarthritis, because “risk of tricyclics is over emphasized”, and “recent reports of fewer falls with tricyclics”, or “they can be used appropriately in low doses for diabetic neuropathy”; thus, this indicator was presented as a positive indicator in Round II.

In general, current clinical guidelines rarely consider the cumulative impact of clinical recommendations, including screening, monitoring and treatment, on individuals with multiple chronic conditions [9, 10]. Thus, there is a lack of scientific evidence on which to build quality indicators for care for people with multiple chronic conditions. Moreover, prior research results demonstrated that over 65% of clinical trials excluded individuals over the age of 70 years, which makes it difficult to understand the impact of medical interventions on these populations [31, 32].

People with diabetes may be prescribed more than a dozen of different classes of drugs to treat diabetes, its complications and other comorbid conditions [33]. Recent research found that poor adherence to drug therapy in people with diabetes is mostly related to the prescribed combination of oral antidiabetic drug therapy and other co-medications [33]. Moreover, disease-specific clinical guidelines may not be appropriate for treating diabetes patients with comorbidities, especially when comorbid conditions are discordant [22, 32].

A small number of outcome indicators defined in this study, especially for care for older adults with discordant concurrent conditions, may be due to the limited scientific evidence linking structure and process to outcomes of care, data limitation issues, or challenges to the accurate measurement because multiple factors contribute to a patient’s health outcomes. There is a need to develop specific outcome measures for older people with multiple chronic conditions to reflect what matters most to patients, which is the effect of all their conditions on their health status [11].

The defined quality indicators form the foundation for future development of quality indicators in the context of various disease combinations. The feedback from the panelists emphasized the importance of developing indicators related to such aspects of care as self-management, patient education, patient-physician relationships, patient’s preferences and goals, and patient adherence to medication. The panelists also highlighted the role of frailty level in developing quality indicators for care for older adults with multiple chronic conditions. Future research is warranted to develop quality indicators that would reflect various aspects of care, including clinical and patient-reported measures, as well as measures of quality of life and efficiency of care.

### Strengths and limitations

Delphi participants were purposefully selected to apply their knowledge and experience to appraise and develop a list of indicators in the context of assessing care of older adults with multiple chronic conditions. Within the Delphi study, panelists did not meet face to face and this enabled a relatively large group of 15 experts from 3 different provinces of Canada to be consulted for the development of a set of indicators. The composition of the expert panel was nearly equal to the non-respondents and can therefore be considered as a good representation of the overall invited panel. In our panel, most of the 15 panelists were in general practice or geriatrics, while the representativeness of the panel was ensured by including clinical pharmacists and a senior methodologist from a quality measurement organization from ac,ross Canada. All experts had been involved in multimorbid patient treatment decisions and/or in a number of research studies or quality improvement activities focusing on patients with multiple chronic conditions.

The main limitations of Delphi methods include purposeful selection of the panelists, attrition rate and non-response bias [34, 35]. The two-phase Delphi study and incorporation of reminder letters helped to prevent attrition. Use of consensus methods in health services research has been criticized in relation to validity [36]. However, evidence suggests that if the expert panel is representative of the area of knowledge, then content validity can be assured [37].

The set of selected indicators includes only those that are amenable to measurement using Ontario administrative data; other potentially meaningful indicators may have been excluded. We were not able to control for severity of chronic conditions due to limitations to clinical sensitivity of administrative data, as well as complexity of administering the survey and running the Delphi process with multiple levels of severity and control. We asked our panel members to consider all conditions as if they were at moderate severity. Meanwhile, the frequency of testing and use of particular medications may depend on the severity of particular illnesses or their combinations. Since the goal of the study was to develop quality indicators based on scientific information and medical advice, patients were not included as panel experts. Future research is warranted to develop quality measures that would reflect what matters most to patients with multiple chronic condition through engaging patients and their family members in the indicator development process. Thus, the developed set of indicators provides a starting point for further investigations that might explore selection of quality indicators for care for older adults with multiple chronic conditions considering severity of illnesses.

The selected set of indicators will be disseminated further at medical conferences and a clinical guidance document will be distributed by the Health System Performance Research Network alongside a link to this manuscript. A performance report on quality of care is planned using the Ontario data and will be disseminated via Health Quality Ontario Quality Standards Branch.

## Conclusions

Quality indicators are important for both quality assessment and quality improvement in healthcare systems. The recommended indicators from this study are not intended to provide a comprehensive tool set for measuring quality of care for older adults with selected disease combinations. Rather, they address clinical aspects of care and can be used as a starting point for further development of quality indicators and use in ambulatory care settings. The recommended indicators are useful for health care providers, managers and policy makers and can be used to evaluate the quality of care for older adults with selected disease combinations in ambulatory care settings. In particular, they can allow health care providers to initiate local quality improvement initiative, systems managers to identify and correct system-wide problems, and policy makers to plan for future systems of care for older adults with diabetes concurrent with selected disease combinations.

## Supporting information

S1 Table Criteria for rating process indicators. (DOCX)

S2 Table Criteria for rating outcome indicators. (DOCX)

S3 Table Results of the Delphi Round I. (DOCX)

S4 Table Results of the Delphi Round II. (DOCX)

S5 Table Categories of patients’ comments. (DOCX)
