# Supplementary material for: Quality indicators for ambulatory care for older adults with diabetes and comorbid conditions: A Delphi study
Source: PLoS One. 2018 Dec 13;13(12):e0208888. doi: 10.1371/journal.pone.0208888 (PMC6292587; doi:10.1371/journal.pone.0208888)
Supplement: S1 Table — (DOCX) [file pone.0208888.s001.docx]

**S1 Table. Criteria for rating process indicators**

| **Meaningfulness** | | | | |
| --- | --- | --- | --- | --- |
| ***This is a meaningful measure of the quality of care we deliver to the patient aged 65 and older diagnosed with this disease combination*** | | | | |
| Not at all  meaningful  (rating=1) | Somewhat meaningful (rating=2) | Moderately meaningful (rating=3) | Very meaningful (rating=4) | Extremely meaningful (rating=5) |
| **Potential for improvements in clinical practices** | | | | |
| ***It is possible to improve the care that impacts this indicator in patients aged 65 and older diagnosed with this disease combination*** | | | | |
| Not at all  possible  (rating=1) | Not at all  possible  (rating=1) | Not at all  possible  (rating=1) | Not at all  possible  (rating=1) | Not at all  possible  (rating=1) |
| **Overall value of inclusion** | | | | |
| **Considering your ratings on all dimensions, rate this process *measure overall for inclusion in the context of this disease combination*** | | | | |
| Do not include (rating=1) | Do not include (rating=1) | Do not include (rating=1) | Do not include (rating=1) | Do not include (rating=1) |
