# Supplementary material for: Quality indicators for ambulatory care for older adults with diabetes and comorbid conditions: A Delphi study
Source: PLoS One. 2018 Dec 13;13(12):e0208888. doi: 10.1371/journal.pone.0208888 (PMC6292587; doi:10.1371/journal.pone.0208888)
Supplement: S2 Table — (DOCX) [file pone.0208888.s002.docx]

**S2 Table. Criteria for rating outcome indicators**

| **Importance** | | | | |
| --- | --- | --- | --- | --- |
| ***This outcome is an important indicator of the quality of care of the patient aged 65 and older diagnosed with this disease combination*** | | | | |
| Not at all  important  (rating=1) | Somewhat important  (rating=2) | Moderately important  (rating=3) | Very important  (rating=4) | Extremely important  (rating=5) |
| **Modifiability** | | | | |
| ***This outcome is potentially modifiable by improvements in patient’s care*** | | | | |
| Not at all  modifiable  (rating=1) | Not at all  modifiable  (rating=1) | Not at all  modifiable  (rating=1) | Not at all  modifiable  (rating=1) | Not at all  modifiable  (rating=1) |
| **Overall value of inclusion** | | | | |
| ***Considering your ratings on all dimensions, rate this outcome measure overall for inclusion in the context of this disease combination*** | | | | |
| Do not include (rating=1) | Do not include (rating=1) | Do not include (rating=1) | Do not include (rating=1) | Do not include (rating=1) |
