# Supplementary material for: Quality indicators for ambulatory care for older adults with diabetes and comorbid conditions: A Delphi study
Source: PLoS One. 2018 Dec 13;13(12):e0208888. doi: 10.1371/journal.pone.0208888 (PMC6292587; doi:10.1371/journal.pone.0208888)
Supplement: S3 Table — (DOCX) [file pone.0208888.s003.docx]

**S3 Table. Results of the Delphi Round I**

| **Quality indicators for care for older adults with diabetes and hypertension** | | | | | | | | | | | | |
| --- | --- | --- | --- | --- | --- | --- | --- | --- | --- | --- | --- | --- |
| **Process Indicator** | | **Meaningfulness** | | **Potential for improvements in clinical practices** | | **Overall value of inclusion** | | | | **Consensus**  **(%)** | | |
|  |  | Median  (min; max) | | Median  (min; max) | | Median  (min; max) | | MADM | |  |  |  |
| ^*^HbA1c testing every 6 months | | 4 (2; 5) | | 4 (2; 5) | | **4 (3: 5)** | | **0.36** | | **80%** | | **Consensus to include** |
| ^**^LDL-cholesterol testing once per year | | 3 (1: 5) | | 3 (1; 5) | | 3 (1; 5) | | 1.11 | |  | | No consensus |
| Eye examination every 1-2 years | | 5 (1; 5) | | 4 (1; 5) | | **5 (1;5)** | | **0.64** | | **86%** | | **Consensus to include** |
| Microalbumin testing once per year | | 4 (2; 5) | | 4 (1; 5) | | 4 (2; 5) | | 0.86 | | 53% | | No consensus |
| Statin therapy | | 3 (1; 5) | | 3 (1; 5) | | 3 (1; 5) | | 1.22 | |  | | No consensus |
| Use of ^***^ACE inhibitors or ARBs | | 4 (2; 5) | | 4 (2; 5) | | **4 (2; 5)** | | **0.86** | | **73%** | | **Consensus to include** |
| Antiplatelet therapy | | 3 (1; 5) | | 3 (1; 5) | | 3 (1; 5) | | 1.00 | |  | | No consensus |
| **Outcome Indicator** | | **Importance** | | **Modifiability** | | **Overall value of inclusion** | | | | **Consensus**  **(%)** | | |
|  |  | **Median**  **(min; max)** | | **Median**  **(min; max)** | | **Median**  **(min; max)** | **MADM** | | |  |  |  |
| Hospital admission rate for diabetes long-term complications | | 4 (3; 5) | | 3 (1; 5) | | 4 (1; 5) | 0.67 | | | 53% | No consensus | |
| Hospital admission rate for diabetes short-term complications | | 4 (1; 5) | | 4 (1; 5) | | 4 (1; 5) | 0.73 | | | 67% | No consensus | |
| Lower-extremity amputation rate | | 4.5 (3; 5) | | 4 (1; 5) | | **4 (1; 5)** | **0.72** | | | **73%** | **Consensus to include** | |
| Cardiovascular mortality rate | | 4 (3; 5) | | 4 (2; 5) | | 4 (2; 5) | 0.83 | | | 67% | No consensus | |
| **Quality indicators for care for older adults with diabetes, hypertension and chronic ischemic heart disease** | | | | | | | | | | | | |
| **Process Indicators** | **Meaningfulness** | | **Potential for improvements in clinical practices** | | **Overall value of inclusion** | | | | **Consensus**  **(%)** | | | |
|  | Median  (min; max) | | Median  (min; max) | | Median  (min; max) | | MADM | |  |  |  |  |
| ^*^HbA1c testing every 6 months | 4 (3; 5) | | 4 (2; 5) | | **4 (3; 5)** | | **0.36** | | **80%** | | **Consensus to include** | |
| ^**^LDL- cholesterol testing once per year | 4 (1; 5) | | 4 (1; 5) | | 4 (1; 5) | | 1.14 | | 60% | | No consensus | |
| Eye examination every 1-2 years | 5 (1; 5) | | 5 (1; 5) | | **5 (1; 5)** | | **0.72** | | **80%** | | **Consensus to include** | |
| Microalbumin testing once per year | 4 (2; 5) | | 4 (2; 5) | | 4 (2; 5) | | 0.79 | | 60% | | No consensus | |
| Statin therapy | 4 (1.5; 5) | | 4 (1.5; 5) | | **4 (1; 5)** | | **0.93** | | **80%** | | **Consensus to include** | |
| Use of ^***^ACE inhibitors or ARBs | 5 (2; 5) | | 5 (2; 5) | | **5 (2; 5)** | | **0.50** | | **87%** | | **Consensus to include** | |
| Beta-blockers therapy | 3.5 (2; 5) | | 4 (2; 5) | | 4 (2; 5) | | 1.00 | | 60% | | No consensus | |
| **Outcome Indicators** | **Importance** | | **Modifiability** | | **Overall value of inclusion** | | | | **Consensus**  **(%)** | | | |
|  | **Median**  **(min; max)** | | **Median**  **(min; max)** | | **Median**  **(min; max)** | | **MADM** | |  |  |  |  |
| Hospital admission rate for diabetes long-term complications | 4 (1; 5) | | 4 (1; 5) | | 4 (1; 5) | | 0.79 | | 67% | | No consensus | |
| Hospital admission rate for diabetes short-term complications | 4 (1; 5) | | 4 (1; 5) | | 4 (1; 5) | | 1.00 | | 60% | | No consensus | |
| Lower-extremity amputation rate | 4 (1; 5) | | 3.5 (1; 5) | | 4 (1; 5) | | 0.72 | | 67% | | No consensus | |
| Cardiovascular mortality rate | 4 (3; 5) | | 4 (2; 5) | | **4 (2; 5)** | | **0.57** | | **80%** | | **Consensus to include** | |
| **Quality indicators for care for older adults with diabetes and osteoarthritis** | | | | | | | | | | | | |
| **Process Indicators** | **Meaningfulness** | | **Potential for improvements in clinical practices** | | **Overall value of inclusion** | | | | **Consensus**  (%) | | | |
|  | Median  (min; max) | | Median  (min; max) | | Median  (min; max) | | MADM | |  |  |  |  |
| ^*^HbA1c testing every 6 months | 3 (1; 5) | | 4 (1; 5) | | 4 (1; 5) | | 0.73 | | 67% | | No consensus | |
| ^**^LDL-cholesterol testing once per year | 3 (1; 5) | | 3 (1; 5) | | 3 (1; 5) | | 1.13 | |  | | No consensus | |
| Eye examination every 1-2 years | 5 (1; 5) | | 4 (1; 5) | | **4 (1; 5)** | | **0.72** | | **87%** | | **Consensus to include** | |
| Microalbumin testing once per year | 4 (2; 5) | | 4 (2; 5) | | 4 (2; 5) | | 0.67 | | 60% | | No consensus | |
| Acetaminophen as first-line therapy | 3 (2; 5) | | 4 (2; 5) | | 3 (2; 5) | | 1.07 | |  | | No consensus | |
| Non-selective ^****^NSAIDs therapy | 3 (1; 5) | | 3 (1; 5) | | 3 (1; 4) | | 0.93 | |  | | No consensus | |
| Cox-selective NSAIDs therapy | 3 (1; 5) | | 3 (1; 4) | | 2 (1; 4) | | 1.00 | | 53% | | No consensus | |
| Non-selective NSAIDs in combination with misoprostol/proton pump inhibitors | 3 (1; 5) | | 3 (1; 5) | | 3 (1; 5) | | 0.93 | |  | | No consensus | |
| **Outcome Indicators** | **Importance** | | **Modifiability** | | **Overall value of inclusion** | | | | **Consensus**  (%) | | | |
|  | Median  (min; max) | | Median  (min; max) | | Median  (min; max) | | MADM | |  |  |  |  |
| Hospital admission rate for diabetes long-term complications | 3 (1; 5) | | 3 (1; 5) | | 3 (1; 5) | | 0.93 | |  | | No consensus | |
| Hospital admission rate for diabetes short-term complications | 4 (1; 5) | | 4 (1; 5) | | 4 (1; 5) | | 1.00 | | 60% | | No consensus | |
| Lower-extremity amputation rate | 4 (1; 5) | | 4 (1; 5) | | **4 (1; 5)** | | **0.93** | | **73%** | | **Consensus to include** | |
| Cardiovascular mortality rate | 4 (2; 5) | | 4 (1; 5) | | 4 (1; 5) | | 0.87 | | 53% | | No consensus | |
| **Quality indicators for care for older adults with diabetes, osteoarthritis and major depression** | | | | | | | | | | | | |
| **Process Indicators** | **Meaningfulness** | | **Potential for improvements in clinical practices** | | **Overall value of inclusion** | | | | **Consensus**  (%) | | | |
|  | Median  (min; max) | | Median  (min; max) | | Median  (min; max) | | MADM | |  |  |  |  |
| ^*^HbA1c testing every 6 months | 4 (1; 5) | | 4 (1; 5) | | 4 (1; 5) | | 0.86 | | 67% | | No consensus | |
| ^**^LDL- cholesterol testing once per year | 3 (1; 5) | | 3 (1; 5) | | 3 (1; 5) | | 1.13 | |  | | No consensus | |
| Eye examination every 1-2 years | 5 (1; 5) | | 4 (1; 5) | | **4 (1; 5)** | | **0.72** | | **87%** | | **Consensus to include** | |
| Microalbumin testing once per year | 3 (2; 5) | | 4 (2; 5) | | 4 (2; 5) | | 1.00 | | 53% | | No consensus | |
| Use of acetaminophen as first-line therapy | 3 (1; 5) | | 3 (2; 5) | | 3 (2; 5) | | 1.00 | |  | | No consensus | |
| ^****^Non-selective NSAIDs therapy | 3 (1; 5) | | 2 (1; 5) | | 2 (1; 4) | | 0.86 | | 67% | | No consensus | |
| Cox-selective NSAIDs therapy | 2 (1; 5) | | 2 (1; 4) | | 2 (1; 4) | | 0.80 | | 60% | | No consensus | |
| Non-selective NSAIDs therapy in combination with misoprostol or proton pump inhibitors | 2 (1; 5) | | 2 (1; 5) | | 2 (1; 4) | | 0.86 | | 60% | | No consensus | |
| Use of tri/tetracyclic antidepressant, benzodiazepine, or monoamine oxidase inhibitors | 3 (1; 5) | | 2 (1; 5) | | 2 (1; 5) | | 1.12 | | 53% | | No consensus | |
| Interval between ^*****^SSRIs and monoamine oxidase inhibitors therapy | 4 (2; 4) | | 4 (2; 5) | | **4 (2; 5)** | | **0.72** | | **87%** | | **Consensus to include** | |
| At least 3 months antidepressant treatment  (acute phase) | 3 (1; 5) | | 3 (1; 5) | | 3 (1; 4) | | 0.93 | |  | | No consensus | |
| At least 6 months antidepressant treatment  (continuation phase) | 3 (1; 4) | | 3 (1; 5) | | 3 (1; 5) | | 1.13 | |  | | No consensus | |
| **Outcome Indicators** | **Importance** | | **Modifiability** | | **Overall value of inclusion** | | | | **Consensus**  (%) | | | |
|  | Median  (min; max) | | Median  (min; max) | | Median  (min; max) | | MADM | |  |  |  |  |
| Hospital admission rate for diabetes long-term complications | 3 (1; 5) | | 4 (1; 5) | | 3 (1; 5) | | 0.93 | |  | | No consensus | |
| Hospital admission rate for diabetes short-term complications (hypo- or hyperglycemia) | 4 (1; 5) | | 4 (1; 5) | | 4 (1; 5) | | 0.86 | | 60% | | No consensus | |
| Lower-extremity amputation rate | 4 (1; 5) | | 3.5 (1; 5) | | 3 (1; 5) | | 1.07 | |  | | No consensus | |
| Cardiovascular mortality rate | 4 (1; 5) | | 4 (1; 5) | | 4 (1; 5) | | 0.67 | | 67% | | No consensus | |
| **Quality indicators for care for older adults with diabetes, osteoarthritis and hypertension** | | | | | | | | | | | | |
| **Process Indicators** | **Meaningfulness** | | **Potential for improvements in clinical practices** | | **Overall value of inclusion** | | | | **Consensus**  (%) | | | |
|  | Median  (min; max) | | Median  (min; max) | | Median  (min; max) | | MADM | |  |  |  |  |
| ^*^HbA1c testing every 6 months | 4 (2; 5) | | 4 (2; 5) | | 4 (2; 5) | | 0.67 | | 60% | | Consensus to include | |
| ^**^LDL- cholesterol testing once per year | 3 (1; 5) | | 3 (1; 5) | | 3 (1; 5) | | 1.08 | |  | | No consensus | |
| Eye examination every 1-2 years | 5 (1; 5) | | 4 (1; 5) | | 5 (1; 5) | | 0.79 | | 80% | | Consensus to include | |
| Microalbumin testing once per year | 3 (2; 5) | | 3 (2; 5) | | 3 (1; 5) | | 0.87 | |  | | No consensus | |
| Statin therapy | 3 (1; 5) | | 4 (1; 5) | | 3 (1; 5) | | 0.89 | |  | | No consensus | |
| ^***^Use of ACE inhibitors or ARBs therapy | 4 (1; 5) | | 4 (1; 5) | | 4 (1; 5) | | 1.13 | | 67% | | Consensus to include | |
| Beta-blocker therapy | 3 (1; 5) | | 3 (1; 5) | | 3 (1; 5) | | 1.07 | |  | | No consensus | |
| Antiplatelet therapy | 3 (1; 5) | | 3 (1; 5) | | 3 (1; 5) | | 0.79 | |  | | No consensus | |
| Acetaminophen as first-line therapy | 3.5 (2; 5) | | 3.5 (2; 5) | | 3 (1; 5) | | 1.07 | |  | | No consensus | |
| Non-selective ^****^NSAIDs therapy | 3 (1; 5) | | 3 (1; 5) | | 2 (1; 5) | | 1.00 | | 53% | | No consensus | |
| Cox-selective NSAID therapy | 3 (1; 5) | | 2 (1; 5) | | 2 (1; 5) | | 1.14 | | 53% | | No consensus | |
| Non-selective NSAID in combination with misoprostol or proton pump inhibitors | 2 (1; 5) | | 2 (1; 5) | | 2 (1; 5) | | 1.22 | | 53% | | No consensus | |
| **Outcome Indicators** | **Importance** | | **Modifiability** | | **Overall value of inclusion** | | | | **Consensus**  (%) | | | |
|  | Median  (min; max) | | Median  (min; max) | | Median  (min; max) | | MADM | |  |  |  |  |
| Hospital admission rate for diabetes long-term complications | 4 (1; 5) | | 3 (1; 5) | | 4 (1; 5) | | 0.93 | | 60% | | No consensus | |
| Hospital admission rate for diabetes short-term complications | 4 (1; 5) | | 4 (1; 5) | | 4 (1; 5) | | 1.00 | | 60% | | No consensus | |
| Lower-extremity amputation rate | 4 (1; 5) | | 4 (1; 5) | | 4 (1; 5) | | 1.00 | | 53% | | No consensus | |
| Cardiovascular mortality rate | 4.5 (1; 5) | | 4 (1; 5) | | 4 (1; 5) | | 0.53 | | 87% | | Consensus to include | |

^*^HbA1c testing=glycated hemoglobin testing

^**^LDL-cholesterol=low-density lipoprotein cholesterol

^***^ACE inhibitors= angiotensin converting enzyme (ACE) inhibitors; ARBs= angiotensin receptor blockers

^****^ NSAIDs therapy=non-steroidal anti-inflammatory drugs

^*****^SSRIs = selective serotonin re-uptake inhibitors
