# Supplementary material for: Quality indicators for ambulatory care for older adults with diabetes and comorbid conditions: A Delphi study
Source: PLoS One. 2018 Dec 13;13(12):e0208888. doi: 10.1371/journal.pone.0208888 (PMC6292587; doi:10.1371/journal.pone.0208888)
Supplement: S4 Table — (DOCX) [file pone.0208888.s004.docx]

**S4 Table. Results of the Delphi round II**

| **Quality indicators for care for older adults with diabetes and hypertension** | | | | | | | | |
| --- | --- | --- | --- | --- | --- | --- | --- | --- |
| **Process**  **Indicator** | **Meaningfulness** | **Potential for improvements in clinical practices** | **Overall value of inclusion** | | | **Consensus**  **(%)** | | |
|  | Median  (min; max) | Median  (min; max) | Median  (min; max) | | MADM |  |  |  |
| ^*^LDL-cholesterol testing once per year | 3 (1: 5) | 4 (1; 5) | 4 (1; 5) | | 1.08 | 53% | | No consensus |
| Microalbumin testing once per year | 4 (2; 5) | 4 (2; 5) | **4 (2; 5)** | | **0.73** | **60%** | | **Consensus to include** |
| Statin therapy | 3 (2; 5) | 4 (2; 5) | 3 (2; 5) | | 1.12 |  | | No consensus |
| Antiplatelet therapy | 3 (1; 5) | 3 (1; 5) | 2 (1; 5) | | 1.08 | 53% | | No consensus |
| ***New indicators*** | | | | | | | | |
| Serum creatinine testing (with eGFR) | 4 (2; 5) | 4 (2; 5) | **4 (2; 5)** | | **0.47** | **87%** | **Consensus to include** | |
| Use of oral hypoglycemic drugs | 4 (2; 5) | 4 (2; 5) | **4 (2; 5)** | | **0.60** | **73%** | **Consensus to include** | |
| Baseline electrocardiography | 3 (1; 5) | 3 (1; 5) | 3 (1; 5) | | 1.27 |  | No consensus | |
| ^**^MRI of head/heart | 1 (1, 4) | 1 (1, 4) | **1 (1, 4)** | | **0.53** | **87%** | **Consensus to reject** | |
| **Outcome indicator** | **Importance** | **Modifiability** | **Overall value of inclusion** | | | **Consensus**  **(%)** | | |
|  | Median  (min; max) | Median  (min; max) | Median  (min; max) | MADM | |  |  |  |
| Hospital admission rate for diabetes long-term complications | 4 (3; 5) | 4 (2; 5) | **4 (1; 5)** | **0.53** | | **67%** | **Consensus to include** | |
| Hospital admission rate for diabetes short-term complications | 4 (3; 5) | 4 (1; 5) | **4 (1; 5)** | **0.73** | | **73%** | **Consensus to include** | |
| Cardiovascular mortality rate | 4 (3; 5) | 4 (2; 5) | **4 (2; 5)** | **0.60** | | **67%** | **Consensus to include** | |
| ***New indicators*** | | | | | | | | |
| All-cause mortality | 4 (2; 5) | 3 (1; 5) | 3 (1; 5) | 0.86 | |  | No consensus | |
| Ocular complications due to diabetes | 4 (3; 5) | 4 (3; 5) | **4 (3; 5)** | **0.47** | | **73%** | **Consensus to include** | |
| Urinary/skin/soft tissue infections | 3 (1; 5) | 3 (1; 5) | 3 (1; 5) | 1.13 | |  | No consensus | |
| **Quality indicators for care for older adults with diabetes, hypertension and chronic ischemic heart disease** | | | | | | | | |
| **Process**  **indicator** | **Meaningfulness** | **Potential for improvements in clinical practices** | **Overall value of inclusion** | | | **Consensus**  **(%)** | | |
|  | Median  (min; max) | Median  (min; max) | Median  (min; max) | MADM | |  |  |  |
| ^*^LDL- cholesterol testing once per year | 4 (1; 5) | 4 (1; 5) | 4 (1; 5) | 0.94 | | 53% | No consensus | |
| Microalbumin testing once per year | 4 (1.; 5) | 4 (1.; 5) | **4 (1.; 5)** | **0.60** | | **73%** | **Consensus to include** | |
| Beta-blockers therapy | 4 (1; 5) | 4 (2; 5) | 4 (1; 5) | 1.00 | | 53% | No consensus | |
| ***New indicator*** | | | | | | | | |
| Antiplatelet therapy | 4 (2; 5) | 4 (2; 5) | **4 (2; 5)** | **0.80** | | **67%** | **Consensus to include** | |
| **Outcome indicator** | **Importance** | **Modifiability** | **Overall value of inclusion** | | | **Consensus**  **(%)** | | |
|  | **Median**  **(min; max)** | **Median**  **(min; max)** | **Median**  **(min; max)** | **MADM** | |  |  |  |
| Hospital admission rate for diabetes long-term complications | 4 (2; 5) | 4 (2; 5) | **4 (2; 5)** | **0.67** | | **67%** | **Consensus to include** | |
| Hospital admission rate for diabetes short-term complications | 4 (3; 5) | 4 (3; 5) | **4 (3; 5)** | **0.53** | | **73%** | **Consensus to include** | |
| Lower-extremity amputation rate | 4 (1; 5) | 4 (1; 5) | **4 (1; 5)** | **0.67** | | **67%** | **Consensus to include** | |
| ***New indicators*** | | | | | | | | |
| Hospital admission for heart failure | 4 (3; 5) | 4 (2; 5) | **4 (2; 5)** | **0.71** | | **67%** | **Consensus to include** | |
| All-cause mortality rate | 4 (2; 5) | 3 (1; 5) | 3 (1; 5) | 0.93 | |  | No consensus | |
| ED visits for diabetes short-term complications | 4 (1; 5) | 4 (1; 5) | **4 (1; 5)** | **0.87** | | **60%** | **Consensus to include** | |
| Bariatric surgery rate | 2 (1; 5) | 2 (1; 5) | **2 (1; 5)** | **1.27** | | **67%** | **Consensus to reject** | |
| **Quality indicators for care for older adults with diabetes and osteoarthritis** | | | | | | | | |
| **Process**  **indicator** | **Meaningfulness** | **Potential for improvements in clinical practices** | **Overall value of inclusion** | | | **Consensus**  (%) | | |
|  | Median  (min; max) | Median  (min; max) | Median  (min; max) | MADM | |  |  |  |
| ^***^HbA1c testing every 6 months | 3 (1; 5) | 4 (1; 5) | **4 (1; 5)** | **0.87** | | **67%** | **Consensus to include** | |
| ^*^LDL-cholesterol testing once per year | 3 (1; 5) | 3 (1; 5) | 3 (1; 5) | 1.06 | |  | No consensus | |
| Microalbumin testing once per year | 4 (1; 5) | 4 (1; 5) | **4 (2; 5)** | **0.73** | | **60%** | **Consensus to include** | |
| Acetaminophen as first-line therapy | 3 (2; 5) | 4 (2; 5) | 3 (2; 5) | 0.87 | |  | No consensus | |
| Non-selective ^****^NSAIDs in combination with misoprostol/proton pump inhibitors | 3 (1; 5) | 3 (1; 4) | 3 (1; 4) | 0.93 | |  | No consensus | |
| ***Modified indicators*** | | | | | | | | |
| Non-selective NSAID therapy  “negative indicator” | 4 (1; 5) | 3 (1; 5) | **4 (1; 4)** | **0.86** | | **60%** | **Consensus to include** | |
| Cox-selective NSAID therapy  “negative indicator” | 3 (1; 5) | 3 (1; 4) | 3 (1; 4) | 0.93 | | 53% | No consensus | |
| ***New indicators*** | | | | | | | | |
| Use of topical NSAIDs | 3 (1; 4) | 3 (1; 5) | 3 (1; 4) | 0.87 | |  | No consensus | |
| Statin therapy | 2 (1, 5) | 3 (1, 5) | 2 (1, 5) | 0.93 | | 53% | No consensus | |
| Use of opioids | 4 (1; 5) | 3 (1; 5) | 3 (1; 5) | 1.00 | |  | No consensus | |
| ^*****^Use of ACE inhibitors | 4 (1; 5) | 3 (1; 5) | 3 (1; 5) | 1.20 | |  | No consensus | |
| Referral for home care | 3 (1; 4) | 3 (1; 5) | 3 (1; 4) | 1.08 | |  | No consensus | |
| **Outcome indicator** | **Importance** | **Modifiability** | **Overall value of inclusion** | | | **Consensus**  (%) | | |
|  | Median  (min; max) | Median  (min; max) | Median  (min; max) | MADM | |  |  |  |
| Hospital admission rate for diabetes long-term complications | 3 (1; 5) | 3 (1; 5) | 3 (1; 5) | 0.93 | |  | No consensus | |
| Hospital admission rate for diabetes short-term complications | 4 (1; 5) | 4 (1; 5) | **4 (1; 5)** | **1.00** | | **73%** | **Consensus to include** | |
| Cardiovascular mortality rate | 4 (2; 5) | 4 (1; 5) | **4 (1; 5)** | **0.67** | | **60%** | **Consensus to include** | |
| ***New indicators*** | | | | | | | | |
| All-cause mortality | 4 (1; 5) | 4 (1; 5) | 4 (1; 5) | 1.20 | | 47% | No consensus | |
| Joint replacement therapy | 3 (1; 5) | 3 (1; 5) | 3 (1; 5) | 1.09 | |  | No consensus | |
| ED visits/hospital admissions for fall | 3 (2; 4) | 3 (2; 4) | 3 (2; 4) | 0.73 | |  | No consensus | |
| **Quality indicators for care for older adults with diabetes, osteoarthritis and major depression** | | | | | | | | |
| **Process Indicators** | **Meaningfulness** | **Potential for improvements in clinical practices** | **Overall value of inclusion** | | | **Consensus**  (%) | | |
|  | Median  (min; max) | Median  (min; max) | Median  (min; max) | MADM | |  |  |  |
| ^***^HbA1c testing every 6 months | 4 (1; 5) | 4 (2; 5) | **4 (1; 5)** | **0.86** | | **60%** | **Consensus to include** | |
| ^*^LDL- cholesterol testing once per year | 3 (1; 5) | 3 (1; 5) | 3 (1; 5) | 0.93 | |  | No consensus | |
| Microalbumin testing once per year | 4 (2; 5) | 4 (2; 5) | **4 (1; 5)** | **0.73** | | **60%** | **Consensus to include** | |
| Use of acetaminophen as first-line therapy | 3 (1; 5) | 3 (2; 5) | 3 (2; 5) | 1.00 | |  | No consensus | |
| Non-selective NSAIDs therapy in combination with misoprostol or proton pump inhibitors | 2 (1; 5) | 3 (1; 5) | 3 (1; 4) | 0.93 | |  | No consensus | |
| At least 3 months antidepressant treatment  (acute phase) | 3 (1; 5) | 3 (1; 5) | 3 (1; 4) | 0.80 | |  | No consensus | |
| At least 6 months antidepressant treatment  (continuation phase) | 3 (1; 4) | 3 (1; 5) | 3 (1; 5) | 1.13 | |  | No consensus | |
| ***Modified indicators*** | | | | | | | | |
| Non-selective ^****^NSAID therapy  “negative indicator” | 4 (1; 5) | 3 (1; 5) | **4 (1; 4)** | **0.86** | | **67%** | **Consensus to include** | |
| Cox-selective NSAID therapy  “negative indicator” | 3 (1; 5) | 3 (1; 4) | 3 (1; 4) | 0.87 | |  | No consensus | |
| Use of tetracyclic antidepressants, benzodiazepines, gaba receptor agonists, or monoamine oxidase inhibitors  “negative indicator” | 3 (1; 5) | 4(1; 5) | **4 (1; 5)** | **0.87** | | **60%** | **Consensus to include** | |
| ***New indicators*** | | | | | | | | |
| ^******^Use of SSRI or SNRI | 2 (1; 5) | 2 (1; 5) | **2 (1; 5)** | **1.01** | | **60%** | **Consensus to reject** | |
| Use of tricyclic antidepressants | 3 (1; 5) | 4 (1; 4) | 4 (1; 4) | 0.79 | | 53% | No consensus | |
| Use of topical NSAIDs | 3 (1; 5) | 3 (1; 5) | 3 (1; 5) | 1.08 | |  | No consensus | |
| Use of opioids | 4 (1; 5) | 4 (1; 5) | 4 (1; 5) | 1.13 | | 53% | No consensus | |
| Referral for home care | 3 (1; 5) | 3 (1; 5) | 3 (1; 5) | 0.89 | |  | No consensus | |
| **Outcome**  **indicator** | **Importance** | **Modifiability** | **Overall value of inclusion** | | | **Consensus**  (%) | | |
|  | Median  (min; max) | Median  (min; max) | Median  (min; max) | MADM | |  |  |  |
| Hospital admission rate for diabetes long-term complications | 3 (1; 5) | 4 (1; 5) | **4 (2; 5)** | **0.67** | | **67%** | **Consensus to include** | |
| Hospital admission rate for diabetes short-term complications (hypo- or hyperglycemia) | 4 (2; 5) | 4 (1; 5) | **4 (2; 5)** | **0.67** | | **67%** | **Consensus to include** | |
| Lower-extremity amputation rate | 4 (1; 5) | 3 (1; 5) | 3 (1; 5) | 0.87 | |  | No consensus | |
| Cardiovascular mortality rate | 4 (1; 4) | 4 (1; 5) | **4 (1; 5)** | **0.40** | | **73%** | **Consensus to include** | |
| ***New indicators*** | | | | | | | | |
| ED visits/hospital admissions for falls | 3 (2; 5) | 4 (1; 5) | 4 (2; 5) | 0.67 | | 43% | No consensus | |
| Hospital admission for depression | 3 (1; 5) | 3 (1; 5) | 3 (1; 5) | 1.20 | |  | No consensus | |
| All-cause ED visits | 4 (1; 5) | 3 (1; 5) | 3 (1; 5) | 0.93 | |  | No consensus | |
| Joint replacement rate | 3 (1; 5) | 3 (1; 4) | 3 (1; 5) | 0.10 | |  | No consensus | |
| **Quality indicators for care for older adults with diabetes, osteoarthritis and hypertension** | | | | | | | | |
| **Process Indicators** | **Meaningfulness** | **Potential for improvements in clinical practices** | **Overall value of inclusion** | | | **Consensus**  (%) | | |
|  | Median  (min; max) | Median  (min; max) | Median  (min; max) | MADM | |  |  |  |
| ^***^HbA1c testing every 6 months | 4 (1; 5) | 4 (2; 5) | 4 (1; 5) | 0.60 | | 73% | Consensus to include | |
| ^*^LDL- cholesterol testing once per year | 3 (1; 5) | 3 (1; 5) | 3 (1; 5) | 1.00 | |  | No consensus | |
| Microalbumin testing once per year | 3 (2; 5) | 4 (2; 5) | 4 (1; 5) | 0.80 | | 60% | Consensus to include | |
| Statin therapy | 3 (1; 5) | 4 (1; 5) | 3 (1; 5) | 0.89 | |  | No consensus | |
| ^*****^Use of ACE inhibitors or ARBs | 4 (1; 5) | 4 (1; 5) | 4 (1; 5) | 0.67 | | 73% | Consensus to include | |
| Beta-blocker therapy | 2 (1; 5) | 2 (1; 4) | 2 (1; 4) | 1.00 | | 67% | Consensus to reject | |
| Antiplatelet therapy | 3 (1; 5) | 3 (1; 5) | 3 (1; 5) | 1.01 | |  | No consensus | |
| Acetaminophen as first-line therapy | 3 (1; 5) | 3 (2; 5) | 3 (2; 5) | 1.06 | |  | No consensus | |
| Non-selective NSAID in combination with misoprostol or proton pump inhibitors | 3 (1; 5) | 2 (1; 5) | 3 (1; 4) | 1.00 | |  | No consensus | |
| ***Modified indicators*** | | | | | | | | |
| Non-selective ^****^NSAID therapy  “negative indicator” | 4 (1; 5) | 4 (1; 5) | 4 (1; 4) | 0.93 | | 60% | Consensus to include | |
| Cox-selective NSAID therapy  “negative indicator” | 3 (1; 5) | 2 (1; 5) | 3 (1; 4) | 1.02 | |  | No consensus | |
| ***New indicators*** | | | | | | | | |
| Use of topical NSAIDs | 3 (1; 5) | 3 (1; 5) | 3 (1; 5) | 0.93 | |  | No consensus | |
| Referral for home care | 3 (1; 4) | 3 (1; 5) | 3 (1; 5) | 0.87 | |  | No consensus | |
| **Outcome Indicators** | **Importance** | **Modifiability** | **Overall value of inclusion** | | | **Consensus**  (%) | | |
|  | Median  (min; max) | Median  (min; max) | Median  (min; max) | MADM | |  |  |  |
| Hospital admission rate for diabetes long-term complications | 4 (2; 5) | 4 (1; 5) | 4 (3; 5) | 0.64 | | 60% | Consensus to include | |
| Hospital admission rate for diabetes short-term complications | 4 (1; 5) | 4 (2; 5) | 4 (2; 5) | 0.60 | | 73% | Consensus to include | |
| Lower-extremity amputation rate | 4 (1; 5) | 3 (1; 5) | 3 (1; 5) | 0.73 | |  | No consensus | |
| ***New indicators*** | | | | | | | | |
| All-cause mortality | 4 (1; 5) | 3 (1; 5) | 3 (1; 5) | 1.07 | |  | No consensus | |
| Joint replacement therapy | 3 (1; 4) | 3 (1; 4) | 3 (1; 4) | 0.93 | |  | No consensus | |
| End-stage renal disease | 4 (2; 5) | 3 (2; 5) | 4 (2; 5) | 0.80 | | 53% | No consensus | |

^*^LDL-cholesterol=low-density lipoprotein cholesterol

^**^MRI of head/heart= magnetic resonance imaging

^***^HbA1c testing=glycated hemoglobin testing

^****^ NSAIDs therapy=non-steroidal anti-inflammatory drugs

^*****^ACE inhibitors= angiotensin converting enzyme (ACE) inhibitors; ARBs= angiotensin receptor blockers

^******^SSRIs = selective serotonin re-uptake inhibitors; SNRI=serotonin norepinephrine reuptake Inhibitor
