# Supplementary material for: Quality indicators for ambulatory care for older adults with diabetes and comorbid conditions: A Delphi study
Source: PLoS One. 2018 Dec 13;13(12):e0208888. doi: 10.1371/journal.pone.0208888 (PMC6292587; doi:10.1371/journal.pone.0208888)
Supplement: S5 Table — (DOCX) [file pone.0208888.s005.docx]

**S5_Table Categories of patients’ comments**

| **Indicator** | **Panelists’ comments categories** |
| --- | --- |
| HbA1c testing every 6-months | ‘The frequency of HbA1c test should be guided by how well a patient controls his/her diabetes’, ‘The regular testing is important and recommended for all patients with diabetes including those with depression. Recommend measuring in the larger diabetes population and reporting by different stratification, including co-morbidity to see if there are any patterns in certain groups and target the interventions towards improving the practices for specific sub groups’ |
| LDL-cholesterol testing once per year | ‘Evidence of statin benefit in the elderly is not closely tied to lipid/cholesterol levels, period’, ‘Very little usefulness in the geriatric population’, ‘Once they are on a statin, they are on a statin. Little need for testing, other than to measure compliance’ |
| Eye examination every 1-2 years | ‘Critical for quality of life. First cataract replacement can reduce falls’, ‘Visual impairment a risk factor for falls and impedes self-care: Early detection of the retinopathy is important as it can be treated’ |
| Microalbumin testing once per year | ‘Potentially good, but might lead to overaggressive treatment and falls’, ‘Low value in elderly patients if stable’, ‘Depends on how well controlled’ |
| Statin therapy | ‘Value could be questioned, especially in the elderly’, ‘The therapy should be very individual and based on patient test results and clinical manifestations. It would be hard to measure the clinical relevance and appropriateness’ |
| ACEI or ARB therapy | ‘ACE therapy is mortality reducing, ARB therapy is not, unless patient has systolic HF’, ‘Important and helpful, if dizziness and falls are asked for to tailor therapy’ |
| Antiplatelet therapy | ‘This would be indicated only if concurrent vascular issues’, ‘Indicator frequency is very patient and situation dependent. Targets for indicator inclusion and assessment should NOT be set as this is not a true measure of the individual physician/patient relationship’ |
| Hospitalization for diabetes long-term complications | ‘Pneumonia, MI, falls, delirium in the elderly are increasingly going to be managed in the home, which as a trend over time affects the value of change in admission rates- all cause admission rate more useful than either long term or short term’, ‘Long-term complications often multifactorial, but an important one’, ‘Avoiding hospitalization is a key outcome’ |
| Hospitalization for diabetes short-term complications | ‘Usually only for type one diabetics, and rare in elderly’, ‘Not the most common reason for ED visits’, ‘This would be very useful to monitor at practice level. Proper outpatient treatment may reduce the incidence and admissions for short term complications’ |
| Lower-extremity amputation rate | “Issues might have started long before, but this is one of the most important things to prevent’ |
| Cardiovascular mortality rate | ‘Especially in depressed patients who also have ischemic heart disease’, ‘Frailty is what dominates prognosis in some. Recent trials do not demonstrate major impact of control on mortality’, ‘This outcome measure may not be specific or attribute to diabetes’ |
| Oral hypoglycemic use | ‘Tricky with those who are diet controlled or metformin intolerant or on other antiglycemics /insulin’, ‘The treatment options are based on different factors that physicians may consider. The results may be very hard to interpret and it may not show the supporting clinical details. In terms of data can be captured only the prescription but not the actual use’ |
| Baseline ECG | ‘Baseline" usually means at time of diabetes diagnosis (or at least that is the recommendation in DM guidelines), so will be difficulty to implement — low score’ |
| MRI head/heart | **‘**Given that most strokes/MIs in diabetics are silent, should we use MRI head (rule out stroke), MRI heart (rule out ischemic disease) to determine which diabetics 'need' statins and ASA, etc.’, ‘Should not be included. It won't be normal regardless of cognitive status’ |
| All-cause mortality | ‘Very little of this attributable to physician care’, ‘It would be hard to attribute mortality rate to diabetes and/or its complications’ |
| Ocular complications due to diabetes | ‘Important functionally’, ‘Most feared complication for patients’ |
| Urinary/skin and soft tissue infections | ‘UTIs are ubiquitous and multifactorial. Skin infects – perhaps’ |
| Beta-blockers therapy | ‘It is important to get a lot of elderly diabetics OFF beta blockers, unless they have systolic heart failure’, ‘Beta-blockers in stable cardiac disease may or may not be standard and depending on patient level characteristics this may be a difficult combination in a patient with diabetes’, ‘Likely depends on how recently acute vascular events occurred’ |
| Bariatric surgery | ‘There is a growing pile of good evidence that bariatric surgery can cure type 2 diabetes, and that it hugely reduces all-cause mortality, even when BMIs are not 'that high'. It might be recommending bariatric surgery to 65-year-old diabetics very soon, especially if they also have hypertension, ischemic heart disease, or a combination’, ‘I would do this if obesity presents - presence of IHD not really the key factor here’ |
| Hospital admissions for heart failure | ‘Any CV admission might be preferable’, ‘Prevalent outcome’, ‘Undertreatment of CAD may lead to HF’ |
| Acetaminophen as first-line therapy | ‘Safest approach”, ‘Effectiveness of acetaminophen has been questioned’, ‘The treatment options are based on multiple factors’, ‘Difficult to measure at scale’ |
| Use of non-selective NSAIDs in combination with misoprostol or proton pump inhibitors | ‘Important but renal issues very important as well, obviously best avoided’, ‘This is not specific for diabetes patients’, ‘Negative indicator’ |
| Use of non-selective NSAIDs | ‘Depends on severity of arthritis rather than concomitant diabetes’, ‘Not in elderly (for more than one week)’, ‘This would be to avoid’, ‘Negative indicator’ |
| Use of cox-selective NSAIDs | ‘Probably to be avoided in case of unsuspected vascular disease’, ‘This would be to avoid’, ‘Negative indicator’, ‘I'm not that happy with drug related process outcomes as family physician - I think we need to focus on all of the non-pharmacologic ways to manage arthritis’ |
| Use of opioids | ‘They shouldn't be on opioids’, ‘The prescription of these medications is based on indications and is not routinely recommended’ |
| Referral for home care | ‘Interesting idea to have this in - I like the idea of trying to capture the community based services in the care of these patients - should it be just the OA + DM or any of the comorbidities? ‘, ‘Depends on frailty and function’ |
| At least 3 months antidepressant treatment (acute phase) | If respond, usual recommendation is treat for a year’, ‘The need to use these drugs depends on the severity of depression’ |
| At least 6 months antidepressant treatment (continuation phase) | ‘Overall the treatments indicators are hard to interpret as there are many other factors that may influence the treatment choice and duration. The need to use these drugs depends on the severity of depression’ |
| Use of tri/tetracyclic antidepressants, benzodiazepines, Z-drugs, or MAOIs | ‘I give SSRIs or SNRIs to just about all of my elderly patients with this triad; I never give tricyclics (mirtazapine is my safer alternative), or benzodiazepines’, ‘This is a negative indicator’, ‘As in "avoid" if possible’, ‘So dependent on circumstances, particularly risks of tricyclics over emphasized’, ‘Split up. TCA less patient issues and wider range of appropriate uses other than depression. e.g. chronic pain’, ‘Have difficulty with this as TCA may be used appropriately in low doses for diabetic neuropathy and may therefore not a negative indicator’, ‘Recent reports of fewer falls with tricyclics!’ |
